# The Relation Between Memory Speed and Capacity: A Domain-General Law of Human Cognition?

**DOI:** 10.5334/joc.83

**Published:** 2019-10-18

**Authors:** Kim Uittenhove, Evie Vergauwe

**Affiliations:** 1 Université de Genève, Faculté de Psychologie et des Sciences de l’Education, Genève, CH

**Keywords:** Working memory, Attention, Memory

## Abstract

This study tests an important and appealing hypothesis that has been around in the fields of cognitive psychology and neuroscience for over 40 years, but that lacks a conclusive empirical test. According to this hypothesis, there is a direct relationship between speed and capacity in working memory. Working memory refers to the ability to retain a small amount of information in a highly accessible state for a short period of time. Across different fields, it has been proposed that the limited capacity of working memory can be understood in terms of time instead of space, such that the amount of information that can be actively maintained corresponds to the amount of information through which one can cycle in a constant and relatively short time-window. Here, we present a study that explicitly and directly tests the speed-capacity hypothesis. In particular, we test (1) the speed-capacity hypothesis in verbal working memory, (2) the speed-capacity hypothesis in visuospatial working memory, and most importantly, (3) whether the same speed-capacity relation holds across verbal and visuospatial working memory, reflecting a domain-general, time-based law of human working memory capacity and, as such, of the complexity of human thought. Overall, our results do not provide any evidence for the existence of a domain-general law. However, unexpected findings related to measuring memory speed (i.e., high prevalence of negative search slopes in the Sternberg task) prevent us from drawing firm conclusions.

Humans can temporarily keep in mind a certain amount of information that is no longer perceptually available in the current environment. This ability is referred to as working memory. The limited capacity of working memory is a critical determinant of human cognitive behavior. Indeed, nearly every cognitive activity requires that some amount of information is kept available over a brief period of time. For example, we rely on working memory when we maintain a phone number, a grocery list, or driving instructions over a short period of time. Furthermore, it plays a crucial role in ongoing cognition, for example when we try to remember the early parts of a sentence until the whole sentence can be heard and integrated in order to understand the meaning, or when we try to remember partial products of an arithmetic problem until the final solution can be calculated. Accordingly, the role of working memory in human cognition has been abundantly documented in a vast range of fields, including reasoning, language, arithmetic, problem solving, and decision-making (e.g., Daneman & Carpenter, 1980; Ormrod & Cochran, 1988; Barrouillet & Lecas, 1999; Süß et al., 2002; Engle, Kane, & Tuholski, 1999; Wilhelm & Oberauer, 2006). Moreover, working memory capacity has been shown to play a major role in cognitive growth in childhood (e.g., Cowan, 2016), cognitive decline in old age (e.g., Salthouse, 1991), individual differences in intellectual abilities (e.g., Conway, Kane, & Engle, 2003), and cognitive deficits observed in neuro-developmental disorders and specific learning difficulties such as attention-deficit hyperactivity disorder (e.g., Rapport, Chung, Shore, & Isaacs, 2001), dyslexia (e.g., Jeffries & Everatt, 2004), and specific language impairment (e.g., Archibald & Gathercole, 2007).

Overall, the limited capacity of working memory is one of the main constraints of the complexity of our thoughts (e.g., Halford, Cowan, & Andrews, 2007; Oberauer, 2009). Therefore, understanding why working memory capacity is limited is key to understanding human cognitive behavior. The current study sets out to test a popular and controversial, yet largely untested, idea: human working memory capacity, expressed as the number of items that one can hold in mind, is closely linked to temporal properties of working memory functioning (i.e., the time needed to search through its contents). Moreover, temporal properties may explain working memory capacity regardless of the domain, whether we maintain verbal or visuospatial information. Testing the domain-generality of working memory limits is one of the priorities in the field.

## Material-based variations in the limits of working memory capacity

A seminal paper by Miller (1956) suggested that people can hold about seven items in mind (e.g., seven unrelated digits, such as 9 1 7 0 3 1 2). Cowan (2001) provided a more nuanced estimate and proposed that about four chunks (i.e., meaningful groups of items) can be held in mind (e.g., 911 007 101 123). However, a variety of empirical findings suggests that working memory capacity is not static in terms of the number of elements that can be held, but that this number instead depends on the specific type of information that is maintained. As an example, Brener (1940) measured how many items could be recalled from immediate memory for different verbal materials and found that participants could recall around eight digits and close to six concrete words (see also Pucket & Kausler, 1984; Crannell & Parrish, 1957, for similar findings). More recently, Alvarez and Cavanagh (2004) found that capacity estimates also significantly differed between various types of visual information, with a span between four and five for colors, and only between one and two for shaded cubes.

The large differences between verbal (e.g., digits and words) and visuospatial information (e.g., colors and shaded cubes), with generally smaller spans for the latter, could be explained by the popular notion of domain-specific mechanisms in working memory. Distinct mechanisms would be responsible for the maintenance of verbal and visuospatial materials, each with their own pool of resources (e.g., Baddeley & Logie, 1999; Shah & Miyake, 1996; Fougnie, Zughni, Godwin, Marois, 2015). It could be assumed that these separate resources have different capacities, thus explaining capacity differences between verbal and visuospatial types of information. However, many researchers disagree with the notion of separate resources for different domains, and instead argue for the existence of central working memory limits (e.g., Cowan, 1999; Engle et al., 1999; Saults & Cowan, 2007; Barrouillet, Bernardin & Camos, 2004). The fact that large capacity differences are not only found between domains but also within each domain casts doubt on a domain-specific explanation, according to which the domain of the material would be the main determinant of the observed capacity. In this paper, we test the idea that a domain-general mechanism is responsible for capacity differences within and also between domains, and that this mechanism relies on temporal properties of working memory functioning.

## Material-based variations in the temporal properties of working memory

Of interest to this idea is the fact that variations have been found in temporal aspects of working memory: the time needed to search through its contents (i.e., memory search). Over fifty years ago, Sternberg (1966) studied how much time participants needed to indicate whether a probe item was present in a small set of memorized elements (Sternberg, 1966, 1969). The rationale was that, given that we need the information in working memory to select the appropriate response, the time taken to arrive at that response will reveal something about the process by which one is searching in memory for that piece of information. In the Sternberg task, short memory lists (e.g., short lists of digits) are presented, followed by a probe item (i.e., a test item requiring a behavioral response to indicate whether it corresponds to one of the list items or not; e.g., button presses). The length of these memory lists is varied and Sternberg discovered that response times were a direct function of list length; response speed slowed down at a rate of about 40 ms per additional item in working memory. This study was one of the first behavioral studies to reveal temporal properties of working memory and the results suggested that working memory might operate in a way that is much more rapid and dynamic than most people think. Importantly, the slope relating response times to list length was found to vary with the type of material to be maintained and searched through. For example, the slope relating response times to list length was 36 ms for digits, 45 ms for nonsense forms, and 56 ms for photographs of faces (Sternberg, 1969). Thus, material-related variations have not only been found in memory capacity, but also in memory search speed. Explaining the relation between these variations could hold the key to understanding the limitations of working memory capacity, with direct repercussions for limitations in higher-order cognition.

## Is there a direct link between material-based variations in both capacity and temporal properties of working memory?

**Figure 1:**
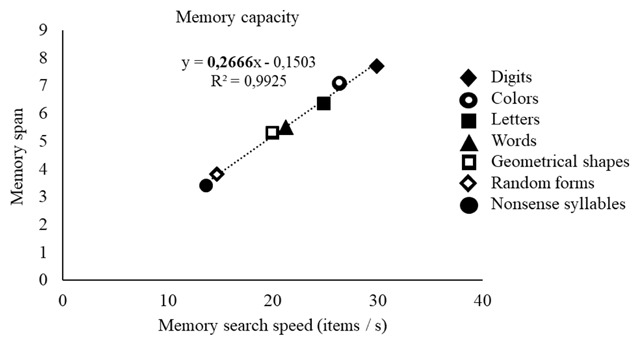
Graph representing the data from Cavanagh (1972). The quantity of items through which one can cycle per second is represented on the x-axis, and the memory span on the y-axis. The figures represent the different materials included in the meta-analysis.

The parallel between material-related variations in memory search speed and in memory capacity did not go unnoticed in the field. In particular, over 45 years ago, Cavanagh (1972) published a meta-analysis examining the link between memory search speed and memory span for different materials. Cavanagh found that faster memory search speeds were associated with larger memory spans. For example, simple stimuli such as digits, which display relatively fast memory search speed (33.4 ms/item), are associated to a relatively high span score (7.7 items in the Cavanagh meta-analysis), relative to more complex stimuli such as nonsense syllables, which display a slower search speed (73 ms/item), and a proportionally lower span (3.4 items). For seven different materials, Cavanagh compared the time needed to search for one item in memory with the proportion of memory space occupied by one item (e.g., one digit takes up a proportion of 0.13 of the total memory space, given that a full memory space contains around 7.7 digits). Cavanagh found that an almost perfectly linear, upwards slope of 243 ms related item speed to item space, which means that it takes 243 ms to cycle through a full memory space. This striking association between capacity and speed across materials and over different studies has been replicated a few times in within-study comparisons as well (e.g., Brown & Kirsner, 1980; Lass et al., 2004; Pucket & Klauser, 1984; Bredenkamp, 2004). For example, Lass et al. (2004) conducted a large study including seven materials (random shapes, geometrical shapes, names of geometrical shapes, colors, names of colors, digits, names of digits) with large samples of German and Chinese participants per material and per task (N ranging from 48 to 144). They replicated the striking association between memory span and memory speed within German and Chinese individuals. The fact that there is a constant relation between speed and capacity suggests that the limits of working memory can be defined in terms of time. Perhaps the amount of information that can be maintained in working memory corresponds to the amount of information through which one can cycle in a constant time-window. We can look at the span for the different materials in the Cavanagh meta-analysis plotted against how many items of each material we can cycle through in one second (see Figure 1). Plotting the data in this way reveals an almost perfectly linear, positive slope of 0.267 relating memory span to memory speed, so that memory span equals the number of items that can be cycled through in 267 ms.

## Importance of understanding the link between the speed of memory search and the capacity of working memory

The idea that the linear relation between speed and capacity reflects the fact that working memory is limited by the number of memories through which we can cycle in a constant time-window, is enticing. Moreover, as pointed out by Vergauwe & Cowan (2014), a similar proposal has been put forward in the neurosciences. In particular, Lisman and Idiart (1995) proposed a model based on brain oscillation patterns. In their model, individual memories are maintained in distinct high-frequency gamma cycles (40 Hz), which are nested in a lower-frequency theta cycle (5 to 12 Hz in Lisman & Idiart, 1995). A theta cycle of 5 Hz would permit to reactivate each memory every 200 ms, by sequentially cycling through the different gamma cycles related to each memory. This model provides a mechanistic view of how working memory capacity would depend on the number of items that can be processed or searched within a given time-window. Moreover, it has been suggested that gamma periods could increase with item complexity (Bahramisharif, Jensen, Jacobs & Lisman, 2018; Atallah & Scanziani, 2009; Sternberg, 2016); materials eliciting slower gamma-cycles would entail that less of these cycles would fit in a theta-cycle of constant length, thereby constituting a basis for finding material-related variations in working memory capacity.

However, despite the appeal of the idea that memory capacity may be related to the speed with which we can search through its content, strong tests of this idea are scarce and some empirical results seem discordant with the idea. In this vein, some researchers find that different types of materials can display very similar memory search rates, yet result in dissimilar spans (e.g., Vergauwe & Cowan, 2014; Pucket & Kausler, 1984). For example, a meta-analysis by Vergauwe and Cowan (2014) reported similar memory search speeds for different types of verbal memoranda, for which capacity usually varies quite a bit (e.g., 36 ms/item for digits, 38 ms/item for letters and 36 ms/item for words, whereas the spans for these materials are 7.7, 6.35 and 5.5, respectively, in the Cavanagh meta-analysis). Results like this are in stark contradiction with the idea that memory search rate and memory span vary together, and cast doubt on the idea of an intrinsic link between memory search rate and memory span. A stronger test of the relationship between memory search speed and working memory capacity is needed.

## Testing for a domain-general relation between memory search speed and capacity

The most convincing evidence would consist in demonstrating that the speed-capacity relation holds in the same way in the verbal and visuospatial domain, showing that this relation can explain capacity differences within and across domains. Overall, empirical results suggest that storage is less efficient for visuospatial material than for verbal material, both in terms of capacity (Alvarez & Cavanagh, 2004), and in terms of more rapid decline of visuospatial information over time (Barrouillet, Uittenhove, Lucidi, & Langerock, 2017; Ricker & Cowan, 2010; Lilienthal, Hale, & Myerson, 2014; Zhang & Luck, 2008). If the speed-capacity relation is a domain-general law of human cognition, the generally lower visuospatial spans should be associated to proportionally slower memory search speeds.

Unfortunately, results for the relation between speed and capacity have been mostly reported for the verbal domain (Pucket & Klauser, 1984; Brown & Kirsner, 1980). Whether and how this relation extends to the visuospatial domain is currently uncharted territory. Although some of the materials used in previous studies did have a visual component (e.g., colors, random forms and geometrical shapes, Cavanagh, 1972; Lass et al., 2004), they could have been easily verbally recoded (e.g., names for colors and geometrical shapes). For example, in the meta-analysis provided by Cavanagh, many of the color and shape materials were easily identifiable (e.g., red, green, blue), came from limited item pools (e.g., 6 to 9 distinct items), and often had to be verbally recalled by giving the corresponding shape or color name (e.g., Brener, 1940), or were extensively familiarized prior to the experiment (Checkosky, 1971). Moreover, in some studies included in the Cavanagh meta-analysis (1972), participants were specifically trained to associate verbal symbols to the visuospatial stimuli (Swanson, Johnson, & Briggs, 1972). Lastly, most of the visuospatial materials used in these studies clearly lacked a spatial component, contrary to many of the visuospatial stimuli used in contemporary lab settings (e.g., for testing visuospatial WM, Kane et al., 2004 used arrows with different orientations, spatial locations in a matrix, and ball movements with different starting points and directions).

There are few studies that may give an indication of the memory search speed that can be expected for visuospatial memoranda. For example, Lecerf and Ribaupierre (2005) found that memory search time for dot locations varied from approximately 1200 ms for two dots, to 1500 ms for four dots, and 2300 ms for five dots, which corresponds to an average increase of 367 ms per item (i.e., a memory speed of 2.7 items/s), which seems much slower than the memory search speed for verbal memoranda. Similarly, Alvarez and Cavanagh (2004) found relatively slow visual search speed for visual stimuli such as shaded cubes (8 items/s) compared to letters (50 items/s) (i.e., in the visual search task participants have to identify a single target stimulus in a display containing distractors). These results, although sparse and from different paradigms, suggest that apart from lower spans, visuospatial materials are also associated to slower search speeds. Before firm conclusions can be drawn, an explicit test of the relationship between speed and capacity within and across verbal and visuospatial working memory is needed. This is exactly what is tested in the present study.

## The present study

Given that a considerable proportion of available data are inconsistent with the speed-capacity relation, and given the relative insufficiency of data for the visuospatial domain, it is of crucial importance to first replicate the striking invariance of the speed-capacity relation within verbal working memory, and second but more interestingly, to generalize it to the visuospatial domain. If we were to find that the same speed-capacity relation holds across verbal and visuospatial working memory, this would suggest that there exists a domain-general, time-based law of human cognition, which determines working memory capacity. Alternatively, if visuospatial and verbal memoranda do not obey the same laws, this would suggest that the differences in capacity commonly found for visuospatial and verbal memoranda cannot be accounted for by a single, domain-general temporal mechanism. In this case, differences in capacity could perhaps be explained by a different use of domain-general resources or by the use of domain-specific working memory resources.

In the current study, we measured memory span and memory search speed within the same individual, with each participant tested on the materials of both the verbal and the visuospatial domains. The material included three types of commonly used verbal memoranda, as well as three visuospatial materials that are often used in contemporary laboratory settings. The materials have been selected from two pilot studies, aimed at finding an optimal spread of span scores for testing a linear relation. We adopted a rigorous study design featuring the same task parameters for the different materials, and for both measures of interest (i.e., memory search rate and memory span). Moreover, recall methods for assessing memory span were closely matched across materials, ensuring maximal comparability of the different spans. In line with the Cavanagh results and interpretation, we should be able to establish a linear relation between speed and capacity in the verbal domain. Moreover, we should find a similar relation in the visuospatial domain. Lastly, if the speed-capacity relation reflects a domain-general mechanism, the same relation should explain results across both domains.

However, it is possible that domain-specific mechanisms like subvocal rehearsal alter memory capacity and memory speed in a way that induces discontinuity between verbal and visuospatial materials, especially given that the existence of such domain-specific maintenance mechanisms is heavily debated for visuospatial memoranda (e.g., Barrouillet & Camos, 2015; Camos, Lagner, & Barrouillet, 2009; Morey, 2018; Vergauwe, Camos, & Barrouillet, 2014; Logie, 2011). If verbal maintenance relies on two distinct maintenance mechanisms, one corresponding to domain-general attention and the other one corresponding to subvocal rehearsal, and if visuospatial maintenance only relies on a single domain-general attentional mechanism, this would have an impact on the relation between speed and capacity. Therefore, if our results were to reveal a discontinuity between the verbal and visuospatial domains, we would proceed by ruling out the domain-specific mechanism of subvocal rehearsal as a cause for this discontinuity, by repeating the verbal tasks under articulatory suppression. If this follow-up experiment were to reveal a single relationship across domains, this would confirm a domain-general law, in addition to verbal but not visuospatial domain-specific resources.

## Method

The present experiment consisted of collecting memory span and memory search speed measures for six different materials (three verbal and three visuospatial). This permitted to 1) test the linear relationship between memory capacity and memory search speed in the verbal domain, 2) extend this relation to visuospatial memoranda, and 3) test the relation across domains. As explained below, in the case of a discontinuity in the relation across domains, a further experiment will aim to rule out subvocal rehearsal as a cause.

### Memory span

**Figure 2:**
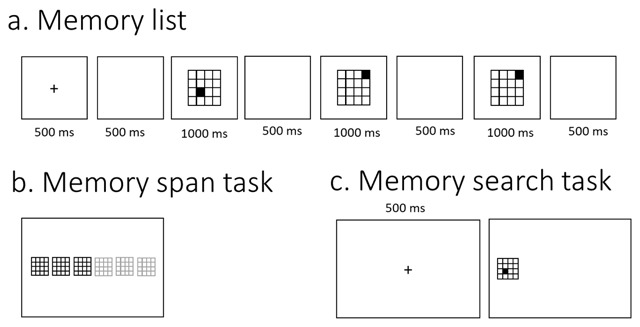
Illustration of the memory span and memory search tests. In both of these tests, participants are first presented with a memory list. In this example, participants have to remember the location of a series of squares in a matrix. In the memory span task, participants subsequently have to click on the correct square in each black matrix, respecting the order of items in the memory list. In the memory search task, participants are presented with a single stimulus, and have to decide whether or not this stimulus was present in the memory list. Different materials will be used in the experiment, all with comparable recall procedures.

In this test, participants were first presented with a memory list (see Figure 2). Participants were instructed to carefully watch and memorize the memory items on the screen, and to reproduce these items in the correct order at the end of each trial. Each trial startedwith a fixation cross presented for 500 ms, followed by a 500 ms blank screen, followed by the series of memory items. Each item was presented for 1000 ms (presentation times in the Cavanagh meta-analysis were in the order of 1–2 seconds), and was followed by a 500 ms blank screen (timings are identical to what was used by Kane et al., 2004). Following the last blank screen, participants had to recall all of the information in the correct order (i.e., order of presentation). For verbal memoranda, they had to enter the items via the keyboard in the correct order and press enter. For this purpose, a number of slots consisting of grey horizontal dashes (i.e., _ _ _) appeared in the middle of the screen. A certain number of these slots, equal to the length of the memory list, was colored in black. Participants had to enter an item (e.g., letter, word, etc.), and then press the spacebar, after which the program switched to the next slot. Before pressing space, participants could still correct their answer. For visuospatial memoranda, participants had to click with the mouse to recall each stimulus. For this, they were presented with a row of grey figures, with each figure containing all of the stimulus possibilities for the task at hand (see Table 2). A number of these figures, again corresponding to the length of the memory list, was colored in black (see Figure 2). For each of these black figures, participants had to recall a single stimulus. Participants could recall a particular stimulus by clicking on the correct possibility. This possibility then changed color to indicate the selection that has been made, and the program switched to the next figure, on which participants could click to recall the next stimulus. We obtained the span for each participant by means of a classic span procedure, gradually increasing the length of the memory list until failure of the participant to recall any of the trials of a particular length. The span scores were calculated by taking the total number of correctly recalled series, divided by the number of trials per length. This method of measuring span is akin to what was used in the studies included by Cavanagh (1972), and is appropriate for our study as well; different materials vary substantially in how many items can be maintained, and a span procedure permitted to detect memory capacity over a complete range of different list lengths. Prior to the memory span test, participants viewed a slide with task instructions and were familiarized with the task through a number of practice trials containing memory lists of two items.

### Memory search

The memory search test involved memory lists with list lengths of two, three, and four items. Participants were instructed to carefully watch and memorize the memory items on the screen, and to subsequently judge whether a probe item was part of this list or not. The procedure for presenting the memory list was the same as for the memory span test (see Figure 2). After presentation of the memory list, a 500 ms fixation cross appeared in the center of the screen. Following this, a probe item was presented in the middle of the left half of the screen (see Figure 2). This placement of the probe item was consistent with the recall task, in which participants recalled the stimuli starting from the left. Moreover, displacing the probe item compared to the memory items forced participants to rely on memory representations instead of on visual aftereffects of the memory items. Participants had to judge as rapidly as possible, and with as few errors as possible, whether or not this item was part of the previously presented memory list. Participants were instructed to have their hand ready on the numerical keypad throughout the trial, and they had to press the 1-key with the right index finger if the probe was not part of the memory list, and the 2-key with their right middle-finger if the probe was part of the memory list. They had to give their response within a 3000 ms delay. There were 30 trials per list length for a total number of 90 experimental trials. As in the original Sternberg studies (e.g., Sternberg, 1966) as well as in other studies that use a similar paradigm (e.g., Chase & Calfee, 1969; Hulme et al., 1999; Pucket & Kausler 1984), trials of different list length were presented in a random sequence. In half of the trials the probe was part of the memory list (i.e., target-present probes), and in the other half of trials, the probe was not present in the memory list (i.e., target-absent probes). Target-present and target-absent trials were randomly intermixed. In the case of a probe that was present in the list, the position of the probe item in that list was randomly chosen, with each position in the list having equal likelihood of being selected. Prior to the memory search test, participants viewed a slide with task instructions and were familiarized with the task through a number of practice trials with memory lists of two, three and four items.

## Pilot data

We conducted two pilot experiments using the memory span task described previously, with the goal of selecting verbal and visuospatial memoranda that create an optimal spread of span. Selecting appropriate materials ensured that, in the subsequent experiments, data points were sufficiently spread out with respect to span measures, to obtain reliable linear regression results. In Pilot Experiment 1, we tested digits, letters, words, and non-words as verbal memoranda. Concerning visuospatial memoranda, we tested locations of squares in a 4 × 4 matrix, locations of squares irregularly placed on the screen, and orientation and length of arrows. In Pilot Experiment 2, we tested additional visuospatial memoranda, including ball movements as well as some novel visuospatial memoranda such as the orientation of lollypops, the size and position of a square on the screen, and the location and orientation of ice-cream cones. The verbal memoranda as well as several of the visuospatial memoranda (except for the novel ones), were adapted from Kane et al. (2004), following as closely as possible the construction of stimuli in their study. Similar to their study, all of our visuospatial tasks sampled items from a pool of 16 different stimuli. A description of the different verbal and visuospatial memoranda can be found in Tables 1 and 2. In general, no items were repeated within a trial unless the span of the participant would exceed the pool of unique possibilities (i.e., a digit span of 10, which is highly exceptional). Concerning words and pseudo-words, and similar to Kane et al. (2004), no items were repeated in the entire memory span task. Note that this entails larger stimulus sets for words and pseudo-words compared to letters or visuospatial memoranda. The main reason for replicating studies that used large stimulus sets for words and pseudo-words (e.g., Kane et al., 2004) is that using restricted stimulus sets for pseudo-words may lead to acquired familiarity over the course of the experiment, which could reduce the differences observed between words and pseudo-words.

**Table 1:** Description of different verbal memoranda used in the pilot experiments.

Verbal memoranda	

**Digits**	Randomly sampled from 0 to 9, presented in uppercase Times New Roman 48 font.
**Letters**	Randomly sampled from 19 consonants (excluding Y and W), presented in uppercase Times New Roman 48 font.
**Words**	Randomly sampled from a set of 312 common French one-syllable words (4 to 6 letters, excluding special characters), based on a French text database (http://www.lexique.org/, developed by Université Savoie Mont Blanc). The items were printed in lowercase Times New Roman 24 font.
**Pseudowords**	Randomly sampled from a selected set of 312 French one-syllable pseudowords (4 to 6 letters, and excluding special characters), matched to the words in terms of frequency of the bigrams involved. The items were printed in lowercase Times New Roman 24 font.

**Table 2:** Description of different visuospatial memoranda used in the pilot experiments.

Visuospatial memoranda		

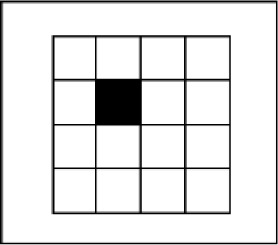	**Locations in a matrix**	Randomly sampled from 16 possible locations in a 4 × 4 matrix consisting of 25 mm × 25 mm squares.
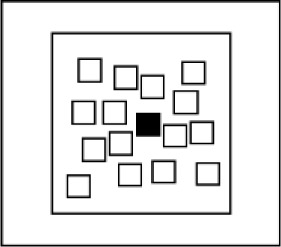	**Irregular locations**	Randomly sampled from 16 possible locations indicated by 25 mm × 25 mm squares that appeared on the screen in a fixed and irregular pattern.
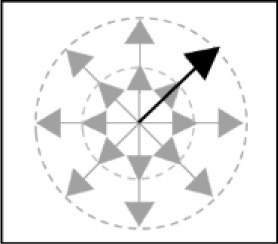	**Arrows**	Randomly sampled from 16 possible arrows, that radiated outwards from the center of the screen and differed in length (2.5 cm or 5 cm) and angle (0°, 45°, 90°, 135°, 180°, 225°, 270°, 315°)
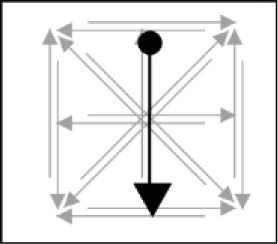	**Ball movements**	Randomly sampled from 16 different movements of a disk (diameter 1,5 cm) within a square box spanning 20 cm on each side. The disk could move on one of 16 possible paths. The possible starting positions corresponded to the corners and the midpoints of the sides of the square. The disk could then move vertically, horizontally or diagonally to the opposite side of the square, in both directions.
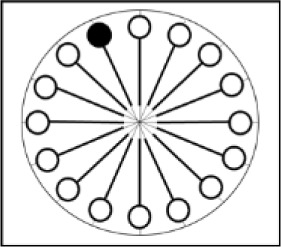	**Lollypops**	Randomly sampled from 16 different lollypops, pointing outwards from the center of the screen, and differing by their angle (from 0° to 337.5° by increments of 22.5°).
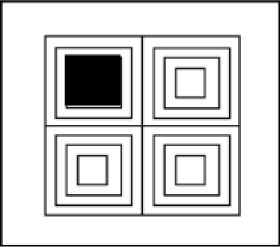	**Squares**	Randomly sampled from 16 different squares, appearing in one of four positions in a 2 × 2 matrix (this matrix spanned 26 cm on each side), and differing in size (sides of 1.5 cm, 3 cm, 5 cm, 8 cm).
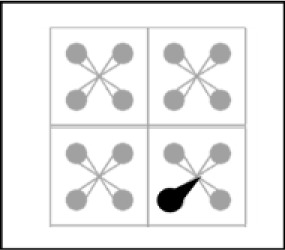	**Ice-cream cones**	Randomly sampled from 16 different cones measuring 2.5 cm in length, appearing in one of four positions in a 2 × 2 matrix (this matrix spanned 16 cm on each side), and differing in orientation (45°, 135°, 225°, 315°).

### Participants and procedure

We tested 12 and 11 participants in Pilot Experiment 1 and 2 respectively (10 females, mean age 21.8, and 8 females, mean age 22.5, respectively). All participants were students recruited from the University of Geneva and received course credit for their participation. They all had normal or corrected-to-normal vision. The pilot experiment was administered in groups of six participants, with each participant seated at a separate booth in the same room. After being welcomed into the room, participants filled out the informed consent for the study (approved by the ethical commission) and a questionnaire about their age, gender, and history of vision or hearing impairments. The entire experiment was fully automatized via a desktop computer, including instructions, training, and test, developed with Tscope5, a C/C++ experiment programming library (developed at Ghent University), and are available on Open Science Framework. The total duration was approximately 60 minutes (Pilot Expt. 1) and 30 minutes (Pilot Expt. 2). The order of the different tasks was counterbalanced with one half of participants completing the tasks in one order, and the other half completing the tasks in the reverse order. Each task started with a slide featuring instructions followed by a training phase containing two trials with memory lists of two items. In the remainder of the pilot experiment, we presented three trials per list length.

### Results

The span was calculated by taking the list length below the minimum list length presented, and adding to this the total number of series correctly recalled divided by the number of trials per list length (i.e., 1 + NbrCorrect/3). The mean spans and standard deviations for the materials tested in both pilot experiments can be found in Table 3 and raw data are available on OSF.

**Table 3:** Mean span and standard deviation (between parentheses) for the different materials tested in both pilot studies.

Memoranda	Span (SD)

*Verbal*	

Pseudo-words	2.06(0.37)
Words	3.39(0.65)
Letters	4.81(1.04)
Digits	5.44(1.23)
***Visuospatial***	

Lollypops	2.61(0.44)
Ball movements	2.79(0.48)
Ice-cream cones	2.88(0.7)
Arrows	3.14(0.72)
Irregular locations	3.19(0.72)
Squares	3.3(0.66)
Locations in a matrix	3.31(0.67)

For the main experiment, we selected three of the four verbal memoranda. To obtain an even spread of data points we chose letters, words and pseudo-words. As can be calculated from Table 3, the difference in span between letters and words, and between words and pseudo-words is of 1.4 and 1.3 items, respectively. Including digits would have led to a less even spread with a difference of only 0.63 items between digits and letters, and 2.03 between digits and words. Then, for the visuospatial memoranda, we wanted to make sure to choose the memoranda that are the farthest apart, because inter-material differences are smaller between different visuospatial stimuli than between different verbal stimuli. This is why we selected locations in a matrix (a classic task) and lollypops (a novel task). The span difference between these two tasks is 0.7 items. Lastly, we selected the ice-cream task, for which performance falls in between the other two tasks, differing by 0.43 items from locations in a matrix, and 0.27 items from lollipops.

**Figure 3:**
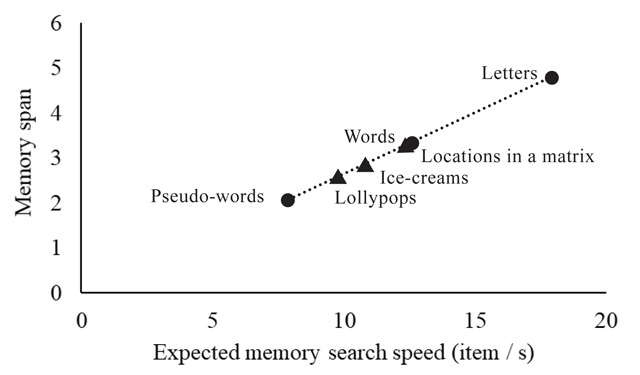
Expected relation between the measured memory span for the selected materials (y-axis), and the memory search speed (items/s) on the y-axis. The circles represent verbal memoranda and the triangles represent visuospatial memoranda.

Based on the measured memory span for the tasks we selected, we can calculate what should be the memory search speed for each material, given the speed-capacity relation extrapolated from the Cavanagh data (memory span = 0.267 * memory speed), and assuming that the same relation holds across domains. The shape of the resulting relation (see Figure 3) is what we should find if the hypothesis of a domain-general law relating memory speed to memory span holds true.

## Main experiment

From the pilot study, we selected letters, words and pseudo-words as verbal memoranda, and lollypops, ice-creams and locations in a matrix as visuospatial memoranda. In the main experiment, we administered to each participant the memory span and memory search test for the three materials of each domain. If there is a fundamental relation between memory capacity and memory speed, and if this relation holds across domains, then we expect to observe (1) a positive and linear relationship between memory span and speed in verbal WM, (2) a positive and linear relationship between memory span and speed in visuospatial WM, and (3) a single positive and linear relation between memory span and speed across verbal and visuospatial WM. Moreover, if the memory span can indeed be calculated by the number of items one can cycle through in 267 ms, the slopes observed in our study should not significantly differ from this value. However, if the data were to reveal a discontinuity between the verbal and visuospatial domain, we planned to refrain from drawing any firm conclusions until having ruled out subvocal rehearsal as a cause in an additional experiment.

### Participants and sampling plan

The data were collected from 36 undergraduate students at the University of Geneva (30 women, mean age 21.8 years, SD 6.6, range 18–53) who were required to have normal or corrected-to-normal vision and hearing. There were no additional selection characteristics. Participants were given the opportunity to earn partial course credit, in case they were enrolled in the psychology department, or a monetary reward of 40 CHF for their participation. After collecting data from 36 participants, we assessed the evidence in favor or against our predictions. If all of our tests would yield a Bayes factor at least 6 times in favor or against our predictions, we had planned to stop testing. In case of insufficient evidence, we had planned to add six more participants, and so forth, until the BFs would reach the specified values, or until a specified maximum of 60 participants would have been tested. In the case where we would have reached 60 participants before arriving at BFs presenting the predefined level of minimum evidence, we planned to interpret those BFs according to the scale proposed by Schönbrodt and Wagenmakers (2018), adjusted from Jeffreys (1961). However, as described below in the section on planned exclusions, applying the exclusion rules as planned resulted in discarding the data from 27 out of 36 participant (i.e., 75%). Based on these numbers, abiding to our planned stopping rule would have entailed testing about 144 participants (3 hours each) to reach a sample size of 36 participants with analyzable data. Going up to 60 participants with analyzable data would have entailed testing up to 240 participants (i.e., 720 hours of testing). This would have been an unreasonably large investment of resources, especially in the light of the limited generalizability of potential findings. Therefore, and in agreement with the editor, data collection stopped entirely after 36 participants.

### Experimental Design

The experiment was divided in two sessions, with one session for the verbal domain and one session for the visuospatial domain, separated by a one-week interval. Each session lasted approximately 90 minutes and involved the memory span and the memory search tests for each of the three materials of a domain. Half of the participants first participated in the verbal session and half first participated in the visuospatial session. For each session, half the participants first performed the memory speed tests for each material, and half the participants began by performing the memory span tests for each material. The three different materials for each domain were presented in three different orders (materials 1–2–3, 3–1–2, or 2–3–1), with each different order administered to one third of the participants.

### Procedure

The experiment was administered in groups of six participants, with each participant seated at a separate booth in the same room. After being welcomed into the room, participants filled out the informed consent (approved by the ethical commission) and a questionnaire about their age, gender, and history of vision or hearing impairments. Participants then performed the memory span tests and the memory search tests (see Figure 2). Before each test for each material, they viewed a slide with instructions and were familiarized with the task in four training trials. For the actual experience, memory search tests used list lengths 2, 3 and 4, with 30 trials per list length, and the memory span tests started at list length 1 (because of low performance for some materials in the pilot studies), with four trials per list length. As in the pilot studies, the entire experiment was fully automatized via a desktop computer, including instructions, training, and test, developed with Tscope5, a C/C++ experiment programming library (Stevens, Lammertyn, Verbuggen, & Vandierendonck, 2006), and are available on OSF.

### Analysis plan: Exclusions

We planned to discard data (1) altogether in case of technical errors (e.g., if equipment were to malfunction), (2) in case a participant correctly recalled less than three memory span trials for one of the materials, or (3) had a correct response rate of less than 60% in the memory search test for a material. Moreover, (4) if the memory search time per item calculated from the memory speed test should be negative across probe-present trials, we planned to exclude the participant from the data.

Concerning (1), in the data we collected from 36 participants, one participant did not complete the memory speed task for one of the materials, for unknown reasons. As a result, this participant had missing memory speed data for one of the materials. The remaining 35 participants completed all the tasks. Regarding (2), all of the participants recalled at least three trials of single-item lists and thus this criterion did not lead to exclusions. Regarding (3) all of the participants had an accuracy of at least 60% for every material and thus this criterion did not lead to exclusions either. More information about the span, speed, and accuracy for different materials can be found in Table 4. The last exclusion criterion (4) proved to be problematic in our sample. Contrary to our expectations, and contrary to the literature (Sternberg, 1966, 2016), many participants had a negative search slope for one out of the six materials, even though the search slopes averaged over participants were in the range of what is reported in the literature (e.g., 37 ms for letters, see Table 4). In the following paragraph, we explain how we tackled this issue in this report.

**Table 4:** Information concerning different materials; including average memory span, average RT (in ms) for probe-present trials, slope relating RT to list length, inverse of slope (items per second), accuracy on probe-present trials, and slope relating accuracy to list length.

		Capacity test		Speed test					

		Span *M*	Span *SD*	RT *M*	RT slope (ms/item)	RT slope *SD*	Inv RT slope (items/s)	Acc *M*	Acc slope (%/item)

*Verbal*	Letters	5.26	*0.87*	852	37	*58*	27	93%	–1.8%
	Words	3.84	*0.64*	840	19	*51*	53	94%	–0.3%
	Pseudowords	2.08	*0.38*	862	38	*50*	26	95%	–1.9%
*Visuospatial*	Matrix	3.56	*0.59*	980	28	*56*	36	93%	–1.6%
	Icecream	2.56	*0.60*	998	74	*49*	14	89%	–3.0%
	Lollypop	2.44	*0.62*	1064	31	*66*	32	80%	–4.2%

A strict application of our exclusion criterion leads to the exclusion of 27 out of 36 participants (i.e., 75% of the total data). In accordance with this exclusion criterion, we will report the results of analysis on a restricted subset of 9 participants, who had exclusively positive search slopes for all materials (i.e., **the planned restricted analysis**). Although the data from these participants meet all our criteria, it must be noted that the scientific validity and generalizability of any conclusions drawn from analyses of such a restricted subset are severely limited.

To address this issue, we present two additional sets of analyses, after consultation with the journal editor. These analyses replicated the planned analyses, but were performed on less restrictive subsets of participants. The first of these additional analyses (i.e., **the less restrictive analysis**) was performed on a subset of participants that have positive search slopes for at least two materials within each domain, while discarding data points that correspond to negative search slopes. Span-speed relationships within a domain can still be calculated for participants that have at least two remaining materials for that domain. Out of 36 participants, 25 meet this new criterion, and were part of the second analysis. The next additional set of analyses was performed on the full sample of participants, with inclusion of negative search slopes (i.e., **the unrestricted analysis**). We reasoned that the high frequency of negative search slopes could indicate that these slopes provide useful information and should be included in any complete analysis investigating the link between search time and memory span. The sample for this analysis contains 35 participants, given that one participant did not complete all of the memory speed tests.

Finally, concerning the reaction time data from the memory search test, we had planned to remove RTs that were abnormally fast (<150 ms). Overall, six trials were discarded following this rule (i.e., .03% of the overall data). We did not apply an upper limit, since participant responses in this task were confined within a 3000 ms window. We had planned to remove the data of participants who had over 20% of their RT data removed in this phase from the analysis altogether. Applying this criterion did not lead to the removal of any participants. Lastly, we intended to replace any participants that were removed in the pre-processing phase before continuing the analysis. However, given that we did not exclude any participant from analysis altogether, we did not replace participants before the planned analysis.

Before analysis, we calculated our measures of interest; memory span and memory speed for each material of each domain. The span scores were calculated for each participant and each material as the number of trials correctly recalled divided by the number of trials per list length (i.e., NbrCorrect/4). The memory speed was calculated for each participant and each material by first calculating the memory search time per item (s/item) as the slope of the linear function of RTs over list length for probe-present trials on which participants responded correctly, and then taking the inverse (items/s). Note however that, when the slope relating probe recognition RT to list length is negative, the inverse of this measure does not necessarily make sense. Indeed, when a participant has a negative slope of –1 ms, we cannot simply conclude that this participant searches through –1000 items per second. This absurdity is accentuated when considering that a participant with a *more* negative slope, such as –100 ms, would be expected to have a *less* negative inverse search rate, corresponding to a search of –10 items per second. Still, as planned, we will report the results for analysis on the inverse of the search time slope, in line with the theoretical model outlined in the introduction. We will tackle the issue regarding the interpretation of those values, in the context of our dataset, by presenting additional analyses, conducted on the directly observed memory search time slopes (e.g., probe recognition RT over list length). We hope that by comparing different analyses, a clear pattern will emerge.

Our first prediction was that we would replicate a positive and linear relationship between memory span and memory speed for the verbal memoranda (letters, words, and pseudo-words). Our second prediction was that we would also have a positive and linear relationship between memory span and memory speed for the visuospatial memoranda (locations in a matrix, ice-creams, and lollypops). Our third prediction was that the same linear relationship would hold over domains. In a first step, each of these three predictions was first tested by calculating for each relation a measure of goodness of fit for a linear relation between memory span and memory speed across materials and averaged over participants. Following Cavanagh (1972), Lass et al. (2004), and other studies that replicated this phenomenon, we calculated a coefficient of determination *R*^2^ as an indicator of how well the averaged memory speed and memory span over materials was explained by a linear relation. We expected to find a coefficient of determination of similar magnitude as these previous studies (.83 in Puckett & Klausler, 1984; >.99 in Cavanagh, 1972; Brown & Kirsner, 1980). In a second step, our three predictions were tested on the individual participant slopes. Data obtained in a within-subjects design by Pucket and Klauser (1984) suggested that the relation between memory span and memory speed also holds within individuals over different materials (with average r = .78, see also Brown & Kirsner, 1980). Similar to these studies, we calculated for each participant the slope relating memory span to memory speed over the three materials for each domain. We then conducted Bayesian one-sample t-tests, to test whether the slopes for each domain were 1) different from zero and 2) positive, in line with our predictions. If we were to find strong evidence in favor of this prediction, additional Bayesian one-sample t-tests would compare the slopes for each domain to the 0.267 value observed by Cavanagh (1972). A final Bayesian paired samples t-test was planned to assess evidence for the prediction of domain-generality by testing whether the individual slopes were the same for the verbal and the visuospatial domain. For all of our tests, we used JASP default Cauchy priors centered around an effect size of zero with a medium scaling constant of 0.707 (JASP Team, 2018). If the results from this analysis would not provide at least moderate evidence (BF > 3 in favor of the null^1^) for the existence of a single positive and linear relationship between speed and capacity across the verbal and the visuospatial domain, we would proceed with a further experiment. This additional experiment would consist of repeating the verbal tasks under articulatory suppression (e.g., repeating the syllables BA BI BOU at a rate of one syllable approximately every 750 ms continuously from the onset of each trial until the end of recall). The results from this follow-up experiment in the verbal domain would subsequently be analyzed and compared to the initial results from the visuospatial domain by following our main analysis plan. The visuospatial slopes from the first experiment would be compared with the verbal slopes from the follow-up experiment with a Bayesian independent samples t-test. This would allow us to test whether a single domain-general relationship between speed and capacity could be observed when the use of subvocal rehearsal is minimized.

## Results of the planned analysis

In what follows, we present the different planned analyses, on three different samples of participants, starting with (A) the planned restricted sample, continuing with (B) the less restrictive sample, and ending with (C) analyses on the unrestricted (full) sample. In each sample, (A), (B) and (C), we tested our three predictions, of (i) a positive relation between memory speed and capacity in the verbal domain, (ii) a positive relation between memory speed and capacity in the visuospatial domain, and (iii) a positive relation between memory speed and capacity across domains. We took different steps in testing each of these predictions, by (1) calculating the linear fit between memory span and the inverse of memory speed (items per sec) averaged over participants, and by (2) performing Bayesian analysis on the individual participant slopes relating memory span to the inverse search slopes (items per sec), as well as (3) the original memory search slopes (sec per item). A full overview in Table 5 allows comparing the different results.

**Table 5:** Summary of evidence from analysis on different subsets of participants. BFs in favor of a positive relation between memory speed and span are indicated in green, BFs in favor of the absence of a positive relation are indicated in red, and anecdotal evidence is indicated in grey.

Sample	N	*Verbal*				*Visuospatial*				*Across domains*			

		Slope	*R*^2^	BF (items/s)	BF (s/items)	Slope	*R*^2^	BF (items/s)	BF (s/items)	Slope	*R*^2^	BF (items/s)	BF (s/items)

Rest.	9	.368	.54	**4.09**	4.31	.310	.31	**5.60**	*0.89*	.288	.55	**3.09**	*1.55*
Interm.	25	–.309	.20	**3.19**	**6.74**	.184	.85	**7.99**	**3.32**	.092	.06	**3.92**	**4.04**
Full	35	.010	.01	**9.26**	**9.9**	.028	.31	**14.02**	*2.23*	.027	.09	**3.92**	**3.43**

***A) Planned restricted sample:** Participants with exclusively positive search time slopes for all materials (N = 9)*.

**Figure 4:**
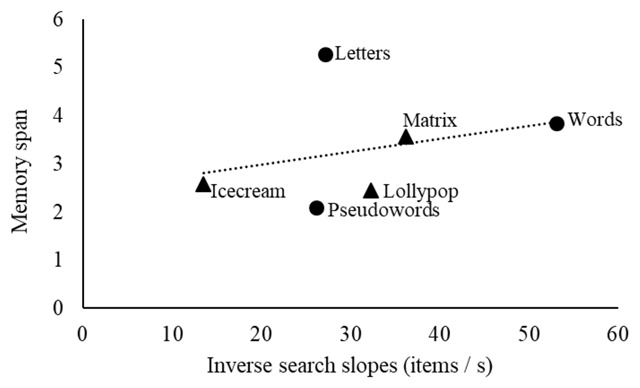
Observed relation between the measured memory span for the six materials (y-axis), and the inverse of the measured memory search slopes (x-axis) in the planned restricted sample (N = 9). The circles represent verbal memoranda and the triangles represent visuospatial memoranda.

1. Linear fit of the averaged sample: The slope relating memory span to inverse search slopes was .368 for the verbal domain (i) with *R*^2^ = .54, .310 for the visuospatial domain (ii) with *R*^2^ = .31, and .288 across domains (iii) with *R*^2^ = .55. A visualization of the relation between inverse search slopes and memory span across the six materials is displayed in Figure 4.
2. Bayesian analysis of individual participant slopes relating memory span to inverse search slopes: Bayesian one-sample tests yielded moderate evidence against slopes that were different from zero and positive in the verbal domain (i), BF_0+_ = 4.09, and in the visuospatial domain (ii), BF_0+_ = 5.60. Bayesian paired-sample t-tests yielded moderate evidence against a difference between slopes from the verbal and the visuospatial domain (iii), BF_01_ = 3.09.
3. Bayesian analysis of individual participant slopes relating memory span to the original memory search slopes: Bayesian one-sample tests yielded moderate evidence against slopes that were different from zero and positive in the verbal domain (i), BF_0+_ = 4.31, and inconclusive evidence in favor of positive slopes in the visuospatial domain (ii), BF_+0_ = 1.12. Bayesian paired-sample t-tests yielded inconclusive evidence against a difference between slopes from the verbal and the visuospatial domain (iii), BF_01_ = 1.55.

**B) Less restrictive sample:** Participants with positive search slopes for at least two materials in each domain (N = 25).

1. Linear fit of the averaged sample: The slope relating memory span to inverse memory search slopes was –.309 for the verbal domain (i) with *R*^2^ = .20, .184 for the visuospatial domain (ii) with *R*^2^ = .85, and .092 across domains (iii) with *R*^2^ = 0.06. However, note that the averages for different materials are not calculated on exactly the same participants, since 16 out of 25 participants had only two materials in one of the domains. To provide information about the degree of overlap between the participants that were included for different materials, we hereby report the number of participants that had a positive search slope for each material. In the verbal domain, 21 participants had a positive search slope for letters, 24 participants had a positive search slope for pseudowords, and 18 participants had a positive search slope for words. In the visuospatial domain, 25 participants had a positive search slope for ice-creams, 21 participants had a positive search slope for lollypops, and 18 participants had a positive search slope for matrices. Note that due to these uneven numbers, comparison with other materials is tentative, especially for those materials yielding data from a small proportion of available participants, such as matrices and words.
2. Bayesian analysis of individual participant slopes relating memory span to inverse memory search slopes: Bayesian one-sample tests yielded moderate evidence against slopes that were different from zero and positive in the verbal domain (i), BF_0+_ = 3.19, and in the visuospatial domain (ii), BF_0+_ = 7.99. Bayesian paired-sample t-tests yielded moderate evidence against a difference between slopes from the verbal and the visuospatial domain (iii), BF_01_ = 3.92.
3. Bayesian analysis of individual participant slopes relating memory span to the original memory search slopes: Bayesian one-sample tests yielded moderate evidence against slopes that were different from zero and positive in the verbal domain (i), BF_0+_ = 6.74, and in the visuospatial domain (ii), BF_0+_ = 3.32. Bayesian paired-sample t-tests yielded moderate evidence against a difference between slopes from the verbal and the visuospatial domain (iii), BF_01_ = 4.04.

**C) Unrestricted sample:** Full sample including negative search time slopes (N = 35).

1. Linear fit of the averaged sample: The slope relating memory span to inverse memory search slopes was .010 for the verbal domain (i) with *R*^2^ = .01, .028 for the visuospatial domain (ii) with *R*^2^ = .31, and .027 across domains (iii) with *R*^2^ = .09.
2. Bayesian analysis of individual participant slopes relating memory span inverse memory search slopes: Bayesian one-sample tests yielded moderate evidence against slopes that were different from zero and positive in the verbal domain (i), BF_0+_ = 9.26, and strong evidence against this effect in the visuospatial domain (ii), BF_0+_ = 14.02. Bayesian paired-sample t-tests yielded moderate evidence against a difference between slopes from the verbal and the visuospatial domain (iii), BF_01_ = 3.92.
3. Bayesian analysis of individual participant slopes relating memory span the original memory search slopes: Bayesian one-sample tests yielded moderate evidence against slopes that were different from zero and positive in the verbal domain (i), BF_0+_ = 9.90, and anecdotal evidence against such an effect in the visuospatial domain (ii), BF_0+_ = 2.23. Bayesian paired-sample t-tests yielded moderate evidence against a difference between slopes from the verbal and the visuospatial domain (iii), BF_01_ = 3.43.

Recall that we initially intended to proceed with a further experiment if our analysis did not provide at least moderate evidence for the existence of a single positive and linear relationship between speed and capacity across the verbal and the visuospatial domain (with BF > 3 in favor of the null regarding the difference between domains). In this further experiment, we had planned to repeat the initial procedure with the addition of articulatory suppression. However, data from the first experiment suggest *absence* of a relation between speed and capacity in both the verbal and the visuospatial domain (see Table 5). Therefore, additional experiments aimed at uncovering the reasons for potential differences in the speed-capacity relation between domains have become unnecessary.

## Discussion

The current study aimed at investigating whether there exists a domain-general law that determines human working-memory capacity as a function of the number of items through which we can cycle in a constant time-window. This domain-general law could potentially account for differences in capacity between the verbal and the visuospatial domain, as well as differences in capacity within each domain. To test the existence of this law, we selected three different verbal materials and three different visuospatial materials, and aimed to measure the memory capacity and memory speed for each material. Whereas memory capacity was measured by a span procedure, memory speed was measured by a Sternberg task, an approach that is widespread in the literature (Sternberg, 1966, 2016; Cavanagh, 1972). As could be expected from the literature, memory span differed in our study between the verbal domain (mean span of 3.7) and the visuospatial domain (mean span of 2.9), as well as within each domain (see Table 4). If the hypothesis of a domain-general law linking memory capacity to memory speed holds true, we should find a linear relation between the spans for these different materials on the one hand, and the measured memory speed for each material on the other hand. More precisely, the higher the measured memory capacity for a material, the higher should be the rate at which we can recycle items from that specific material, as illustrated in Figure 3.

We employed a within-subjects approach with 36 participants to test this prediction on the global as well as on the individual level. However, many of our participants (i.e., 27 out of 36) exhibited a negative search rate for at least one of the six materials for which they were tested, a surprising finding that we will explore further in the discussion. Consequently, applying our planned exclusion criteria led to the selection of a very small subset of the total sample, consisting of only 9 out of 36 original participants. To deal with this issue, as specified in the data analysis section, we tested our predictions in three different subsets of participants. One subset included the planned restricted sample, including only those participants who exhibited exclusively positive search rates (N = 9), another less restricted subset included additional participants who had a minimum of two positive search rates within each domain (N = 25), and the final full sample included all participants regardless of search rate (N = 35). Overall, a coherent pattern emerges from analyses on these different subsets.

On the global level, results from the planned restricted sample do not testify to a systematic domain-general law relating memory capacity and memory speed, nor do they testify to a domain-specific law relating speed and capacity within the verbal or within the visuospatial domain of working memory. As can be seen in Table 5, when considering the relation between average span and average inverse memory search slope for each material, linear fit (*R*^2^) never reaches the high values found in previous research (>.99 in Cavanagh, 1972); the coefficients of determination range from 0.31 in the visuospatial domain to 0.54 for the verbal domain, and 0.55 across domains. The corresponding slopes range from 0.288 across domains to 0.310 in the visuospatial domain, and 0.368 in the verbal domain, which may be compared with the 0.267 slope from previous research (Cavanagh, 1972; Lass et al., 2004). From these rather modest values for linear fit, we can conclude that materials associated with higher memory capacity have a tendency to be associated with higher memory speed as well, but this tendency is far from systematic and does not testify to the existence of a cognitive law. A comparison of the predicted relation between speed and capacity (Figure 3) and the observed relation (Figure 4) perfectly illustrates the observed lack of systematicity, which is problematic for the hypothesis of a cognitive law.

Furthermore, still focusing on the results of the planned restricted sample, but turning to the individual data instead of the global averages, we mostly find evidence against the existence of a positive relation between memory capacity and memory speed (see Bayes factors in Table 5). However, recall that the subset of participants involved in this analysis was very limited, containing only 9 out of 36 original participants. One must tread carefully when attempting to interpret and generalize the results of such a small sample, partly because of the limited number of participants, and partly because the resulting conclusions only hold for a small proportion (i.e., 25%) of the tested sample. Our further analyses aimed at addressing this issue of generalizability.

These further analyses, conducted on less restricted subsets, confirmed the absence of evidence for a domain-general, or even a domain-specific, law relating memory capacity to memory speed. As can be seen in Table 5, the coefficients of determination for a linear relation between speed and capacity are even lower in the less restrictive and unrestricted subsets (all *R*^2^ < .31). There is one exception in the less restrictive sample, with *R*^2^ = .85 in the visuospatial domain for a slope of .184, but the existence of a relationship between speed and capacity in the visuospatial domain in this subset of the sample is not confirmed by analysis on individual participants’ slopes (BF of 7.69 against a positive relation between speed and capacity). More generally, Bayesian analyses on individual participants’ slopes, in all subsets of the sample and in all domains, yield evidence against the existence of a positive relation between memory speed and capacity.

Thus, overall, our results do not provide any evidence for the existence of a domain-general law, where memory speed explains memory capacity. Moreover, our results do not provide any evidence for the existence of domain-specific law, where memory speed explains memory capacity within the verbal domain and/or within the visuospatial domain of working memory. However, in spite of these negative results, we cannot definitively conclude that such a law does not exist.

One important aspect of our data precludes such a strong conclusion, and merits additional exploration to gain further understanding: The unexpected high prevalence of negative search slopes. Usually, research papers report search slopes averaged across participants, and do not present detailed analysis of the distribution of these slopes (e.g., Sternberg, 1966; Cavanagh, 1972). We note that the average slopes for different materials obtained in the present study are in fact comparable to the values reported in the literature. For example, we can consider letters, which is one of the most widely studied materials that is also included in the present study. We see in Table 4 that the observed search slope for letters in our study is 37 ms, very close to the slope of 40.2 ms reported in the Cavanagh (1972) meta-analysis, especially when considering that the individual studies included in this paper reported slopes ranging anywhere between 29 and 65 milliseconds. Therefore, the average values obtained in our study are no cause for concern. However, in order to confirm the existence of a cognitive law, we deemed it relevant to take analysis one step further and check individual search slopes. It is only during this deeper examination of the data that a problematic omnipresence of negative slopes was revealed, that would surely have stayed hidden if we had only reported average slopes. One could argue that these aberrant results are related to the inclusion of novel types of stimuli in the current study. Analysis of the prevalence of negative slopes show that 9 participants (25%) had a negative slope for letters, 12 had a negative slope for words (33%), 7 had a negative slope for pseudowords (19%), 11 had a negative slope for matrices (31%), 2 had a negative slope for ice-creams (5%), and 9 had a negative slope for lollipops (25%). Although there is some variation in the prevalence of negative slopes across materials, these numbers show that negative slopes are not confined to one or two specific materials but exist across a wide range of materials and across domains. Similarly, when exploring whether negative slopes are characteristic of a specific type of participant, we find that only 9 participants had no negative slopes, whereas 9 had a single negative slope, 11 had two negative slopes, 5 had three negative slopes, and a single participant had four negative slopes. Thus, it would appear that negative slopes appear in a wide range of participants, for a wide range of materials, and devoid of any systematic pattern.

One particular recurrent issue regarding the Sternberg task is the serial position curves related to the probe item for probe-present trials. According to Sternberg (1969), this serial position curve is flat, indicating an exhaustive scanning of the entire memory list. However, in the last 50 years many researchers have observed serial position curves that are not flat, suggesting a different memory search process (Burrows & Okada, 1971; Monsell, 1978; Nee & Jonides, 2008). For example, most recently, Vergauwe and Langerock (2017) clearly showed an RT-advantage for the recognition of probe items that appeared as the last item in the memory list, which is at odds with the notion of a serial search through the list. Note that in the present study, the serial position of probes in the memory list was randomly determined with uniform probability across positions, a procedure commonly used in the typical Sternberg experiment (e.g., Sternberg, 1969). Therefore, in some cases, there may be a higher number of last-presented (LP) probes for a specific list length. One could imagine that a higher occurrence of LP-probes for longer lists would lower the average RT for these list lengths, consequently overturning supposedly positive relations between RT and list length. To examine whether this was the case in our study, we conducted additional analyses. For lists of 4 items, the theoretical frequency of LP-probes is around 3.75 items out of 15. We collected all 63 cases where a participant, for a specific material, had more than 4 LP-probes. Of these cases, 30% exhibited a negative slope relating RT to list length, compared to 21% for the remaining instances. Bayesian contingency analysis showed anecdotal evidence for a null effect, regarding the existence of a difference (BF_01_ = 2.3). A similar logic can be followed for the frequency of LP-probes for short lists of 2 items. Less LP-probes for those lists could increase average RTs for short list lengths. In those cases with less than 7 out of 15 LP-probes, 31% of search slopes were negative, compared to 21% in the remaining cases. Again, Bayesian contingency analysis showed anecdotal evidence for a null effect (BF_01_ = 2.0). Given these results, it is unlikely that the distribution of LP-probes between list lengths accounts for the prevalence of negative slopes in our sample.

**Figure 5:**
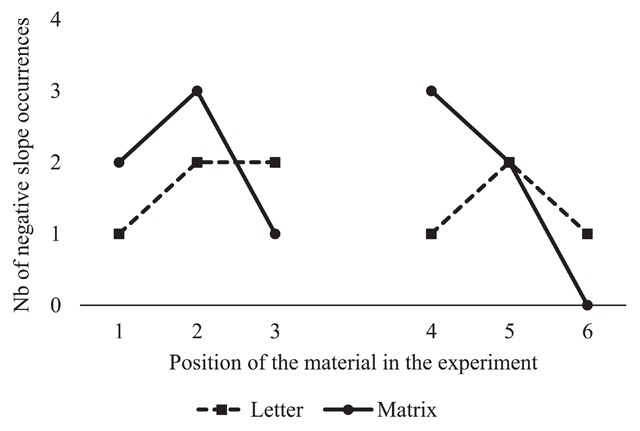
Number of occurrences of negative search slopes for letters and matrices over blocks within the first session (1, 2, 3) and the second session (4, 5, 6).

Another possibility would be that the occurrence of negative search slopes reflects strategic adaptations in participants^2^. More precisely, participants may use a slower and more accurate strategy for small and easily maintained lists of 2 items (e.g., scanning the items in memory), and a faster and less accurate strategy for larger lists of 4 items that are less easily maintained (e.g., recognition of the probe item based on activation strength). If participants use a different and faster strategy for larger lists than for small lists, this could lead to a reversal of list length slopes. We may see the footprints of such strategic adaptations in two ways. First, if these strategic adaptations developed during the task, we would see an increase of the prevalence of negative search slope as the task progresses. Remember that participants completed two sessions of 90 minutes each, with three materials per session. Figure 5 illustrates the occurrence of negative search slopes for letters and matrices, which are two of the most commonly used memoranda in the literature for the verbal and the visuo-spatial domain, respectively. The pattern of data in Figure 5 does not show any systematic increase in negative search slopes over time for letters and matrices within or between sessions. Therefore, if negative search slopes are due to strategic adaptations, these adaptations are likely already present at the beginning of the experiment. Second, strategic adaptations involving a quicker but less thorough strategy for larger lists would likely lead to lower accuracy than the use of slower more thorough strategies. For letters, 9 participants with a negative search slope exhibited an average accuracy of 88.9% (*SD* 11.8%) for trials with lists of 4 items, compared to 94.0% (*SD* 6.2%) for the 26 individuals with non-negative search slopes. An independent samples Bayesian one-sided t-test indicated anecdotal evidence at best in favor of lower accuracy with lists of 4 items for participants with negative search slopes (BF_+0_ = 1.8). For matrices, 11 participants with a negative slope exhibited an average accuracy of 89.4% (*SD* 6.5%) for lists with 4 items, compared to 92.9% (*SD* 6.8%) for the 24 individuals with non-negative search slopes. An independent samples Bayesian one-sided t-test indicated anecdotal evidence at best in favor of lower accuracy with lists of 4 items for participants with negative search slopes (BF_+0_ = 1.33). Although analyses are inconclusive, due to relatively small and unequally distributed numbers of observations, the pattern of results is not inconsistent with the idea that some participants use less accurate but faster strategies with larger lists. If strategic adaptations are indeed the case, they would pose a problem to any study using the Sternberg paradigm, since we observed negative slopes from the beginning of our experiment and the slope averaged over participants is close to what is reported in the literature (e.g., 37 ms for letters). Therefore, it seems unlikely that the high prevalence of negative search slopes in our study is caused by specific details of our study design, such as the inclusion of several materials within a session and/or the inclusion of several sessions.

Thus far, the mystery of negative slopes largely remains. The occurrence of such slopes causes concern from a theoretical point of view. None of the current models that aim to account for performance in a Sternberg-paradigm would predict a prevalence of negative slopes ranging between 19% and 33% for verbal materials. A glance at the distribution of slopes for each material in appendix A suggests that negative slopes are more than just noise, and often reach values far below zero. These illustrations also show a lot of variation between individuals in the size of the slopes. Because of this variation, the paradigm with which we intended to measure memory speed did not detect any differences between materials, contrary to the memory span paradigm. For example, Bayesian paired-sample t-tests found no evidence for a difference between the three verbal materials, in the measures obtained with the memory speed paradigm (BF_10_ in favor of a difference ranging between .183 and .742). Future research may need to focus on the distribution of the slopes relating list length to RT, given that this particular relation has recently been established as one of the most important benchmarks for validating models of working memory (see benchmark A 1.2 in Oberauer et al., 2018).

In conclusion, the present study did not find any evidence for the existence of a cognitive law that relates memory capacity to memory speed. However, our study also raises serious concerns about the utility of the Sternberg paradigm for measuring memory speed. At least in our study, the cognitive axiom (e.g., Oberauer et al. 2018; Sternberg, 2016) according to which probe recognition RT necessarily increases with the length of the memory list does not hold true. Thus, instead of revealing a law of human cognition, our study shakes the very foundations of a cognitive principle that we often take for granted.

## Data Accessibility Statement

The data and scripts corresponding to the experimental protocol can be found at the following online depository: https://osf.io/r7vw3/.

## Additional File

The additional file for this article can be found as follows:

10.5334/joc.83.s1 Appendix A. Normalized distributions of the slopes relating RT to list length (in ms) for each material.

## Footnotes

Note that for deciding whether a follow-up experiment was needed, we proposed a lower threshold of evidence than for the participant sampling plan. We considered it unwarranted to conduct any follow-up experiment exploring differences between verbal and visuospatial memoranda if moderate evidence (BF > 3) for the null hypothesis were to be observed (i.e., moderate evidence against a difference between verbal and visuospatial data).

We thank an anonymous reviewer for this suggestion.
