# Supplementary material for: The Relation Between Memory Speed and Capacity: A Domain-General Law of Human Cognition?
Source: J Cogn. 2019 Oct 18;2(1):41. doi: 10.5334/joc.83 (PMC6798901; doi:10.5334/joc.83)
Supplement: Appendix A. — Normalized distributions of the slopes relating RT to list length (in ms) for each material. [file joc-2-1-83-s1.pdf]

Appendix A: Normalized distributions of the slopes relating RT to list length (in ms) for each material.

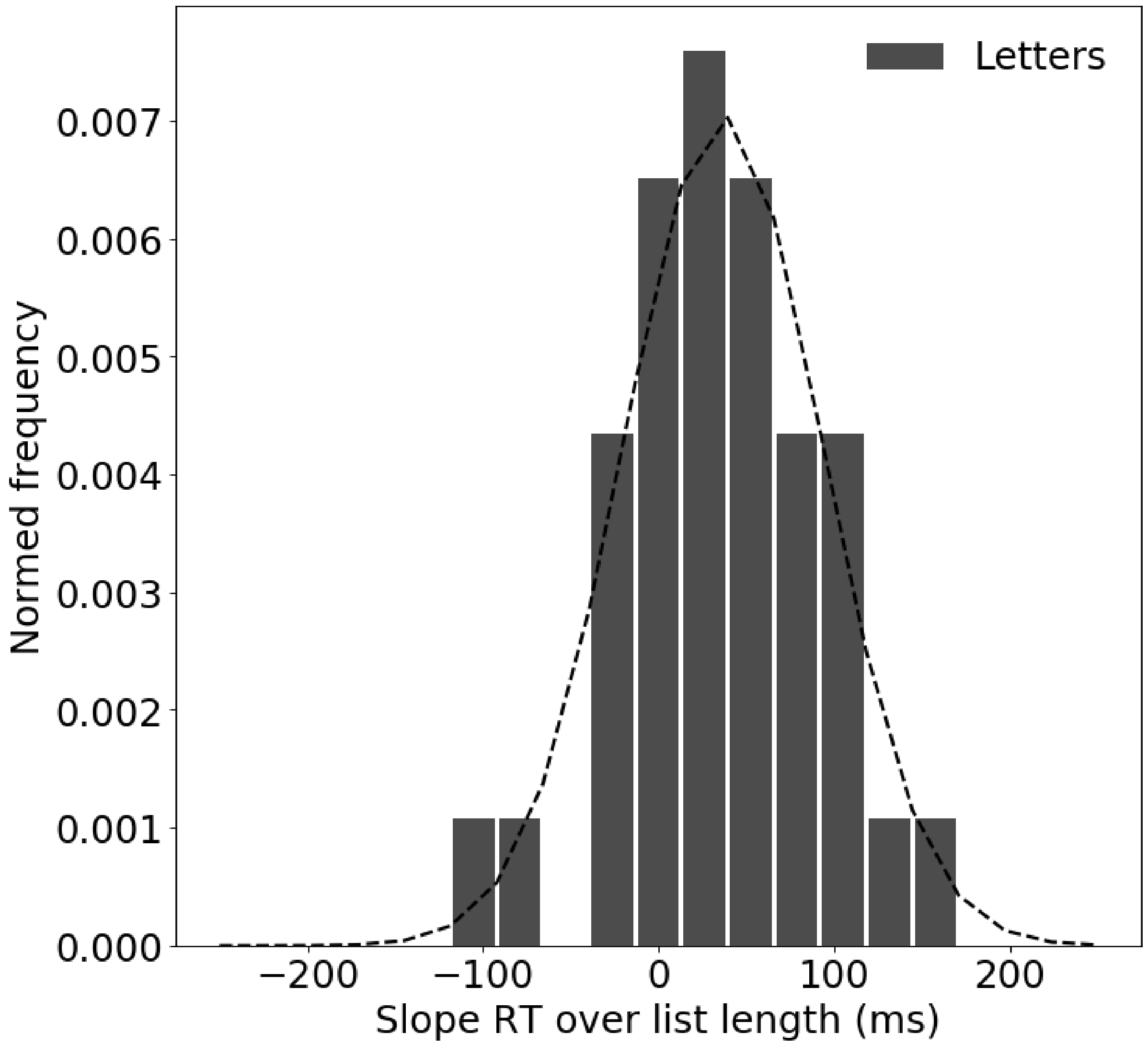

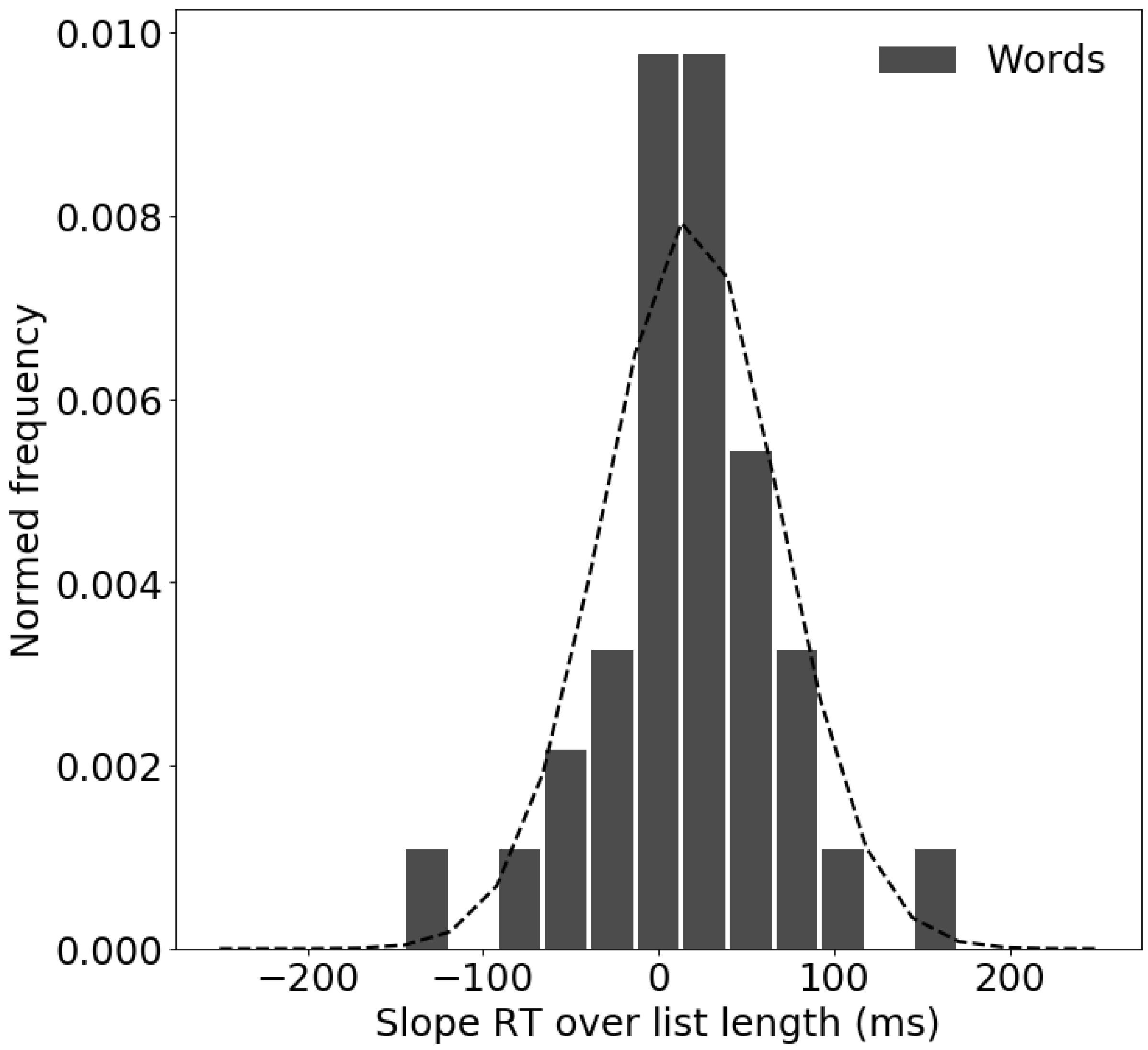
Words
Normed frequency
0.010
0.008
0.006
0.004
0.002
0.000
−200
−100
0
100
200
Slope RT over list length (ms)

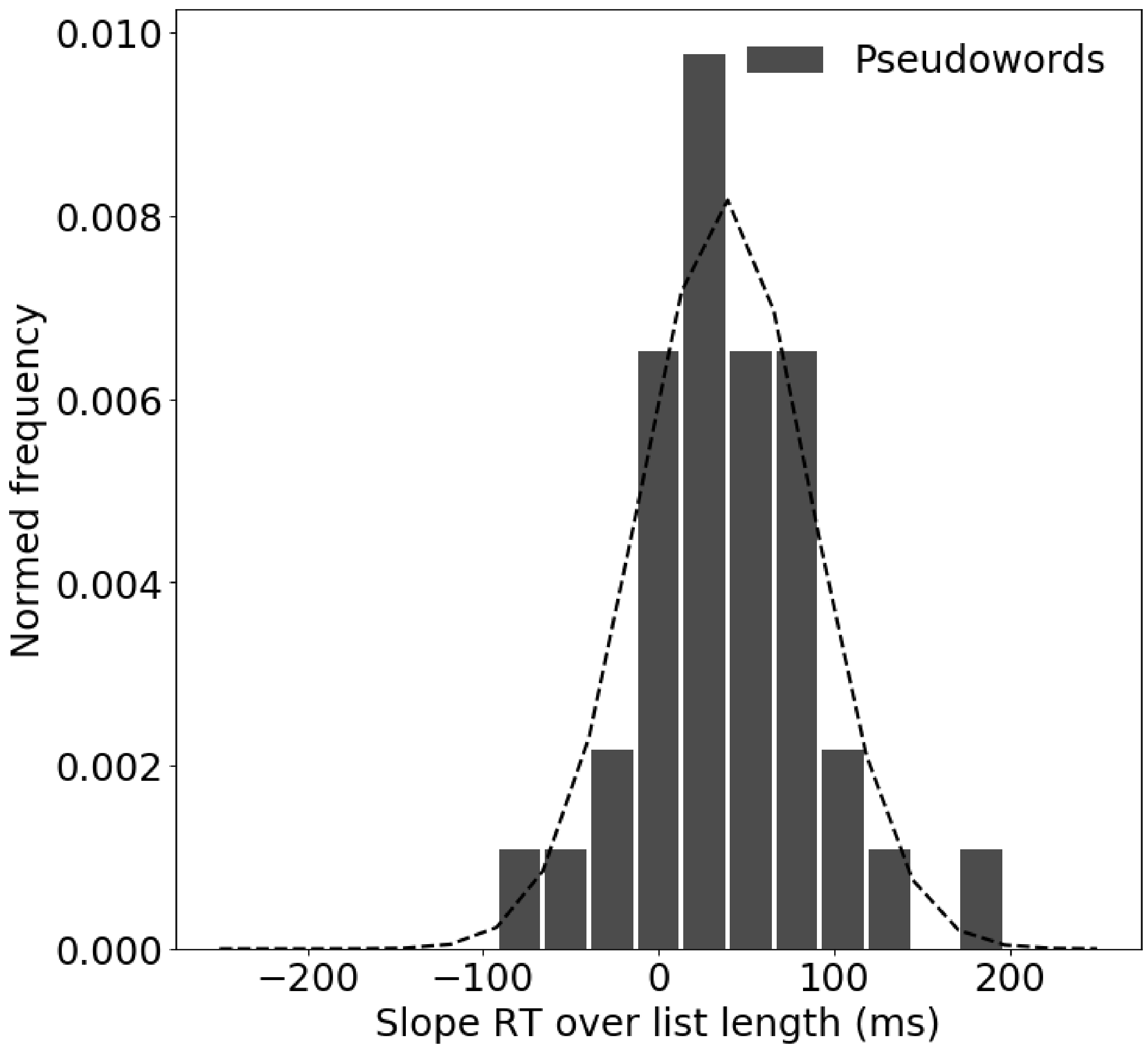
Pseudowords
Normed frequency
0.010
0.008
0.006
0.004
0.002
0.000
−200
−100
0
100
200
Slope RT over list length (ms)

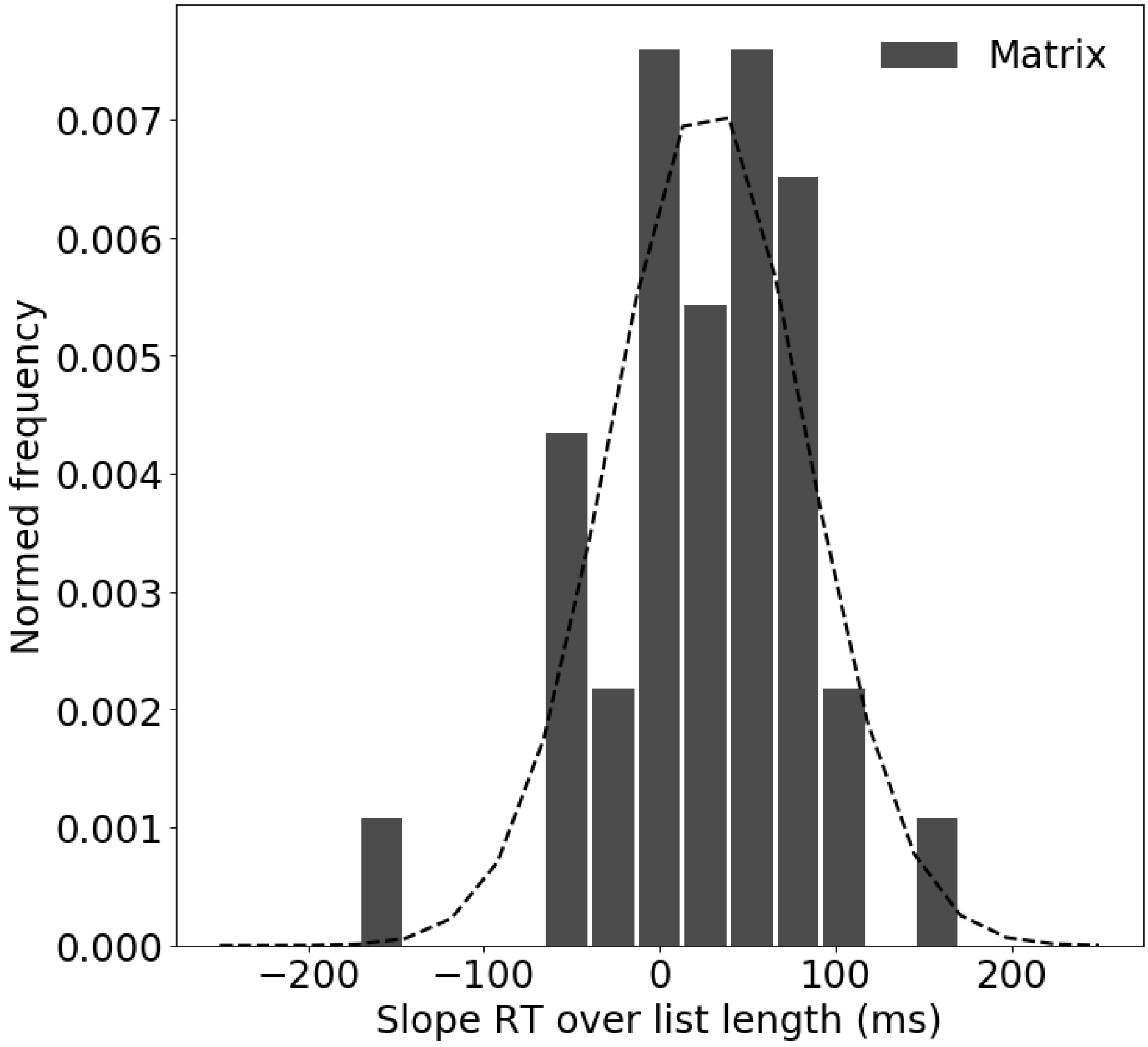
Matrix
Normed frequency
0.000
0.001
0.002
0.003
0.004
0.005
0.006
0.007
−200
−100
0
100
200
Slope RT over list length (ms)

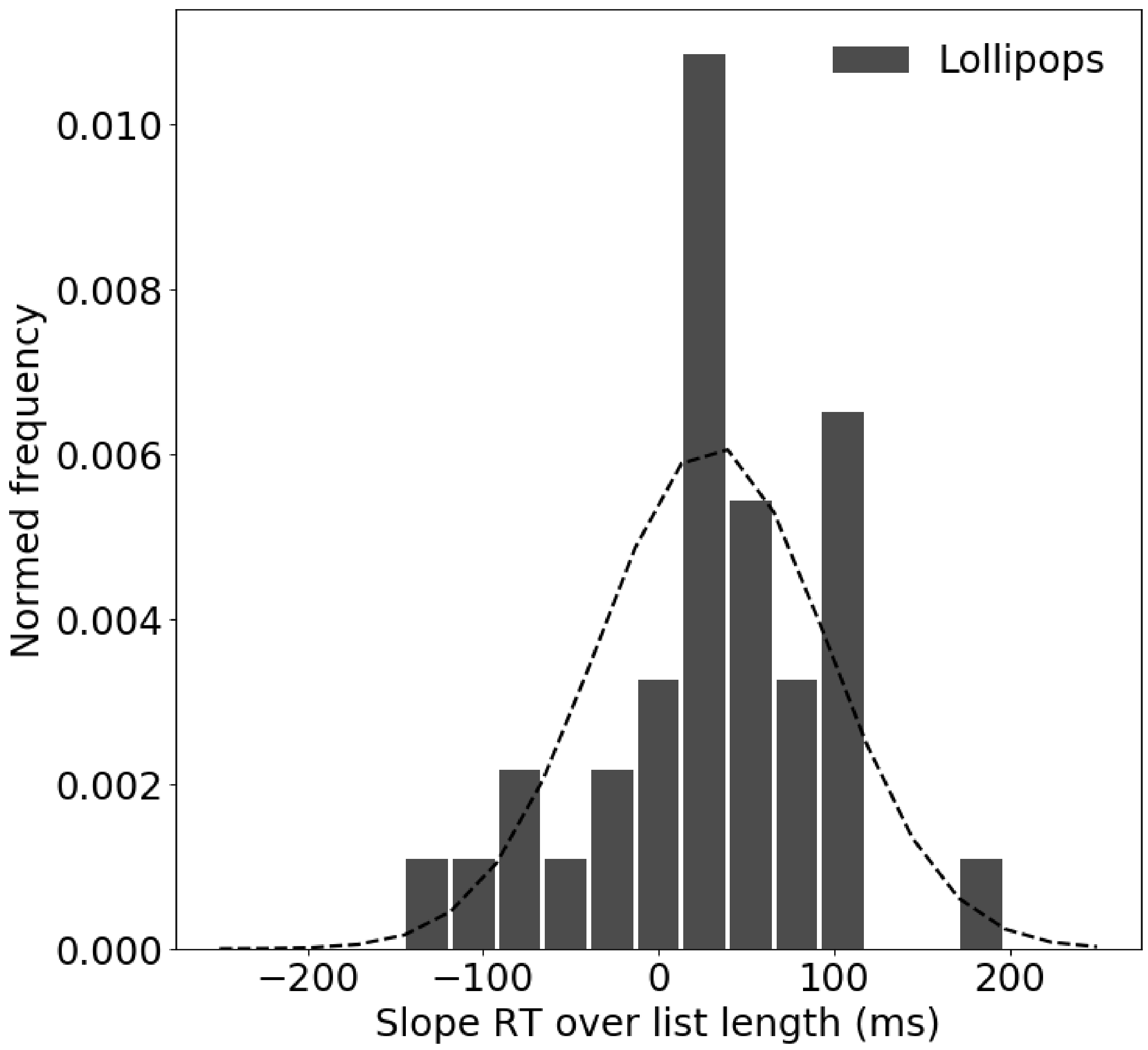
Lollipops
0.010
0.008
0.006
0.004
0.002
0.000
Normed frequency
−200
−100
0
100
200
Slope RT over list length (ms)

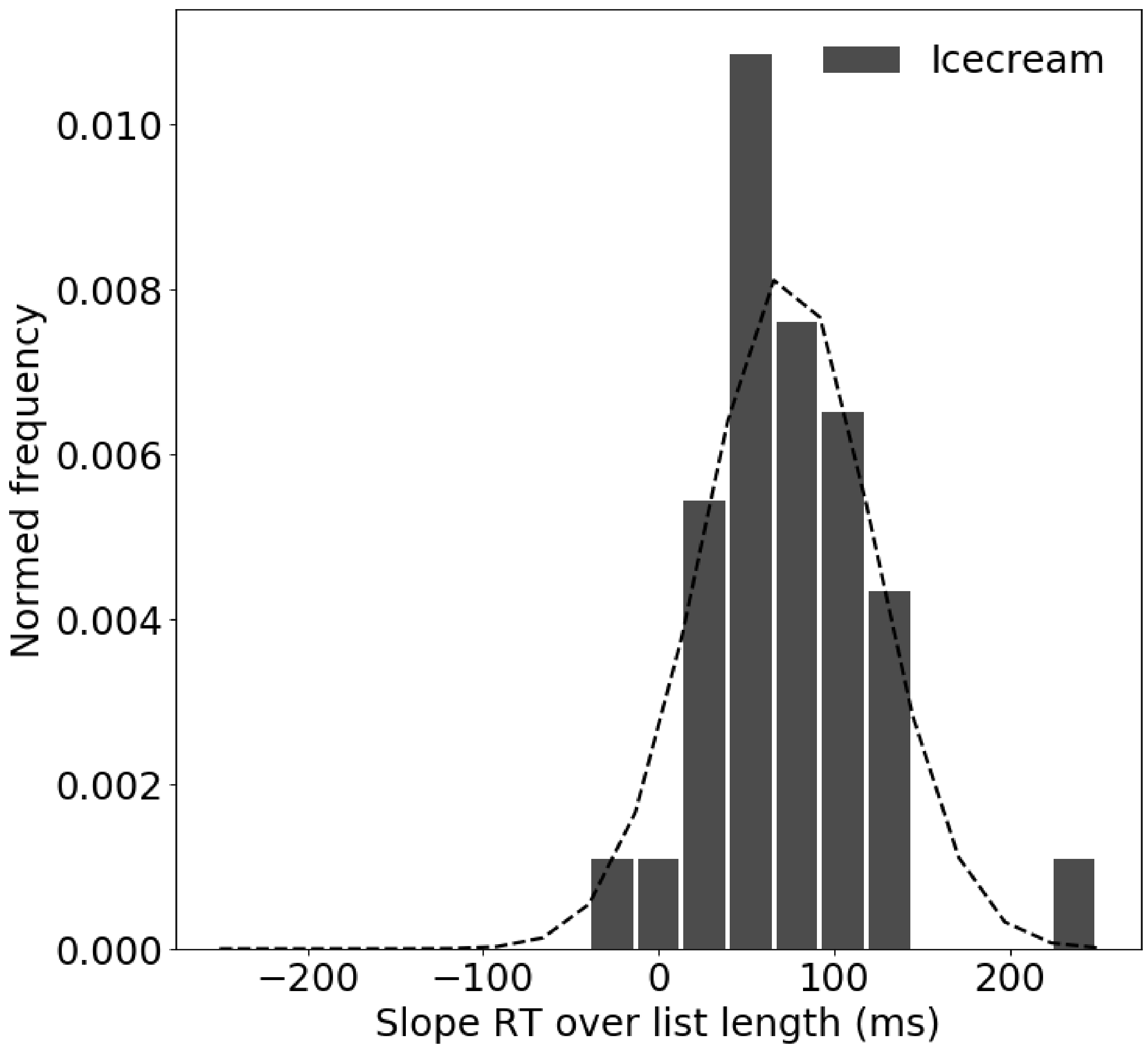
Icecream
Normed frequency
0.000
0.002
0.004
0.006
0.008
0.010
−200
−100
0
100
200
Slope RT over list length (ms)
